# The Role of Claudins in the Pathogenesis of Dextran Sulfate Sodium-Induced Experimental Colitis: The Effects of Nobiletin

**DOI:** 10.3390/biom14091122

**Published:** 2024-09-04

**Authors:** Asmaa Al-Failakawi, Aishah Al-Jarallah, Muddanna Rao, Islam Khan

**Affiliations:** 1 Department of Biochemistry, College of Medicine, Kuwait University, P.O. Box 24923, Safat 13110, Kuwait; asma.alfailakawi@grad.ku.edu.kw (A.A.-F.); aishah.aljarallah@ku.edu.kw (A.A.-J.); 2 Departments of Anatomy, College of Medicine, Kuwait University, P.O. Box 24923, Safat 13110, Kuwait; muddanna.rao@ku.edu.kw

**Keywords:** colitis, claudins, inflammation, tight junctions, dextran sulfate sodium, myeloperoxidase

## Abstract

Background: The pathogenesis of inflammatory bowel diseases such as ulcerative colitis and Crohn’s disease is not well understood. This study investigated the roles and regulation of the claudin-1, -2, -3, and -4 isoforms in the pathogenesis of ulcerative colitis, and the potential therapeutic effects of nobiletin. Methods: Colitis was induced in rats by administering dextran sulfate sodium [DSS] in drinking water for seven days. Animals were treated daily with nobiletin [oral, 60 mg/Kg body weight] and studied in four groups, C [non-colitis control], D [DSS-induced colitis], CN [nobiletin-treated non-colitis control], and DN [nobiletin-treated DSS-induced colitis]. On day seven, the animals were sacrificed, and colonic tissues were collected and analyzed. Results: Both macroscopic and microscopic findings suggest the progression of colitis. In the inflamed colon, claudin-1 and -4 proteins were decreased, claudin-2 increased, while the claudin-3 protein remained unchanged. Except for claudin-1, these changes were not paralleled by mRNA expression, indicating a complex regulatory mechanism. Uniform β-actin expression along with consistent quality and yield of total RNA indicated selectivity of these changes. Nobiletin treatment reversed these changes. Conclusions: Altered expression of the claudin isoforms -1, -2, and -4 disrupts tight junctions, exposing the lamina propria to microflora, leading to electrolyte disturbance and the development of ulcerative colitis. Nobiletin with its anti-inflammatory properties may be useful in IBD.

## 1. Introduction

Inflammatory bowel diseases [IBDs] such as Crohn’s disease [CD] and ulcerative colitis [UC] are chronic inflammatory conditions of the gastrointestinal tract, affecting individuals in the prime of life. These conditions are associated with a significant reduction in colonic epithelial resistance due to a complex protein structure known as a tight junction which is present between intestinal epithelial cells [1,2,3,4,5]. The tight junctions are formed by a large family of small integral membrane proteins known as claudins, which function as a barrier against the luminal contents and microflora [1,2,3,4,5]. There are 26 different isoforms of claudins which show tissue and cell-selective expression and regulation. Besides their role in physical barrier formation, they also regulate paracellular permeability, cell signaling, and epithelial cell polarity [6,7]. Claudin isoforms-1, -3, and -4 participate in barrier formation, and the isoform -2 regulates colonic paracellular permeability [8,9]. Claudin-2 is expressed in both the villus and crypt cells of the small intestine, whereas in the colon, it is restricted to undifferentiated crypt cells. Claudin-3 and -4 isoforms are predominantly expressed in the distal colon [10].

These proteins play a crucial role in maintaining intestinal homeostasis under normal physiological conditions. For example, altered expression of claudin-1 has been reported in human IBD conditions [11,12,13]. An increased expression of claudin-2 is associated with enhanced permeability, diarrhea, and inflammation; however, animals over-expressing or under-expressing the claudin-2 isoform exhibit different responses to the induction of colitis [3]. Significantly reduced expression of claudin-2 is reported to impair barrier functions and paracellular permeability in ulcerative colitis and Crohn’s disease [14]. Furthermore, both increased and decreased expression patterns of claudin-3 have been reported in colonic biopsies from IBD patients [5,14,15,16]. The claudin-4 isoform is expressed in epithelial cells and goblet cells in the colon. In addition, claudin-4 gene knockout [-/-] mice develop colitis-like symptoms associated with increased intestinal permeability [17]. These variations in the expression of claudins highlight their complex regulation in IBD conditions and require further investigations to establish a regulatory mechanism [8]. Given the reported inconsistencies in the expression of claudin isoforms, it is essential to investigate the roles and regulation of the major colonic claudin isoforms in IBD.

IBD conditions are treated using chemicals which are associated with serious side effects; therefore, plant products such as nobiletin have also received attention. Nobiletin, a plant flavonoid which is extracted from lemon peels, possesses anti-inflammatory and anti-oxidative properties [18,19,20]. Nobiletin ameliorates symptoms of IBD; however, whether it is associated with a reversal in the expression of claudin isoforms remains to be established. Therefore, the primary objective of this study was to investigate the roles of the major colonic claudin isoforms -1, -2, -3, and -4 in the acute phase of colitis induced by DSS in male Sprague-Dawley rats and their reversal by nobiletin treatment.

In this study, colitis was induced in male Sprague-Dawley rats by giving them free access to DSS in drinking water for seven days. This model is extensively used to investigate the pathogenesis and drug development for IBD conditions. We focused on investigating the regulation of claudins in the early stage of the development of colitis over a seven-day period. We report that DSS causes alterations in the expression of claudin-1, -2, and -4 isoforms, but not claudin-3, in the colon. These changes are reversed by nobiletin. We suggest that these aberrations in protein expression lead to a leaky gut, increasing the exposure of lamina propria to microflora and hence the development of colitis.

## 2. Materials and Methods

### 2.1. Development of Colitis

Male Sprague-Dawley rats weighing 100–150 gm were maintained by the Animal Care Facility, College of Medicine, Kuwait University, Kuwait. The animals were housed in an air-conditioned room maintained at 20 °C with a 12-h cycle of light/dark and managed following standard ethical guidelines issued by the Animal Care Facility, College of Medicine, Kuwait University, Kuwait. They were allowed free access to feed and water. This study was approved by the Health Sciences Research Ethics Committee, with approval number [23/VDR/EC].

Colitis was induced by dextran sulfate sodium [DSS, 36,000–50,000 MW, MP Biomedicals Inc., Santa Ana, CA, USA] administered orally through drinking water. The animals were given free intake of a freshly prepared 6% DSS solution in autoclaved water for the first 3 days, followed by a switch to a 3% DSS solution in drinking water for an additional 4 days, until day 7 [21]. An aqueous suspension of nobiletin [60 mg/kg BW, Catalogue # CFN98726, China] was administered daily once by oral gavage, starting 2 days before the induction of colitis until 2 h before sacrificing the animals on day 7 post-induction. The dose of nobiletin was selected based on published reports [18,22,23]; this dose was effective and did not produce any ill effects in the non-colitis control group used in this study. The day that DSS administration began was considered day 0. Body weight, food and water intake, and urine output were recorded daily until day 7.

The animals were divided into four groups: the non-colitis control [C], the nobiletin-treated non-colitis group [CN], the colitis group [D], and the nobiletin-treated DSS colitis group [DN]. On day 7, after recording their body weights, the animals were sacrificed by cervical dislocation without any anesthesia; colonic tissues were taken out, cleaned with ice-cold phosphate-buffered saline [PBS], and used in various experiments.

### 2.2. Myeloperoxidase Activity

Using a standard colorimetric method, myeloperoxidase activity [MPO] was measured in the colonic tissues to confirm colitis [24,25]. Tissue lysates were centrifuged at 1000× *g* for 10 min at 4 °C, and the supernatants were collected to measure MPO activity using an O-dianisidine chromogenic substrate [Sigma, Macclesfield, UK]. The activity was expressed as units per minute per mg tissue [25]. An enzyme unit is defined as the number of nmoles of H_2_O_2_ [Sigma, UK] converted into water at an ambient temperature.

### 2.3. Body and Colon Weights

Body weight was recorded daily, starting at two days before inducing colitis by DSS until day 7 post-induction of colitis. Day 0 was defined as the day when DSS administration began. A difference in their body weights between day 0 and day 7 was expressed as percentage changes with respect to their body weights on day 0. The total length and weight of each colon were recorded on day 7 post-sacrifice, and the weight of each colon is expressed as gm/cm colon from the test conditions.

### 2.4. Disease Activity Index

The disease activity index [DAI] was calculated utilizing the following well-established score system [22] and was used to characterize colitis:

- Weight loss [0 point = none, 1 point = 1–5% weight loss, 2 points = 5–10% weight loss, 3 points = 10–15% weight loss, and 4 points = >15% weight loss].
- Stool consistency/diarrhea [0 points = normal, 1–2 points = loose stools, and 3–4 points = watery diarrhea].
- Bloody stool [0 points = no bleeding, 1–2 = slight bleeding, and 3–4 points = gross bleeding].

The DAI, calculated as the total sum of these scores [0 to 12], was defined as the total DAI score, where ‘0’ represents unaffected and ‘12’ indicates severe colitis [22].

### 2.5. Food Efficiency Ratio

Active inflammation in IBD affects nutrient absorption and metabolism, which are indicated by a change in the food efficiency ratio [FER]. This parameter correlates with disease activity and serves as an additional index to assess the severity of the disease. The FER was calculated by dividing the change in body weight per gram of food intake on day 7 under the test conditions.

### 2.6. Hematoxylin and Eosin Staining

Colonic segments were cleaned with ice-cold PBS, fixed in paraformaldehyde, and embedded in paraffin solution to prepare tissue blocks. Thin sections [5 μm thickness] were cut using a microtome and placed onto the coated-glass slides to stain with hematoxylin and eosin dyes [Sigma], as described earlier [26]. Histological changes were estimated using the stained tissue sections under the microscope [Olympus BX 51 TF, Tokyo, Japan] and photographed using an attached camera [Olympus DP 71 camera, Tokyo, Japan].

### 2.7. Mucin Staining

Goblet cells secrete mucins onto the gastrointestinal epithelium and form a defense layer against microflora and food antigens. Mucins were stained using an alcian blue solution [1% alcian dye, pH 2.5] following the method used earlier [26]. The stained sections were viewed under a microscope [Olympus BX 51 TF] and photographed using a camera [Olympus DP 71 camera] attached to the microscope.

### 2.8. Histology Score

Histological scores were calculated using the following criteria [27] and used to characterize colitis in the present model:

**Table:** 

Features	Changes	Score
Hyperplasia	<25%	1
	26–35%	2 or 3
	36–50%	3 or 4
	>51%	4 or 5
Goblet cell loss	<20%	1
	21–35%	2 or 3
	36–50%	3 or 4
	>51%	4
Erosion		1–4
Irregular crypts		1–5
Crypt loss		1–5

Histological changes were characterized by the loss and deformity of crypt cells, the loss of goblet cells, hyperplasia, and epithelial erosions in the tissue sections from the current model of colitis.

### 2.9. Expression of Claudin Proteins

Immunofluorescence microscopy was employed to examine the expression of claudins in the colonic tissues, and the data were validated by ECL Western blot analysis.

### 2.10. Immunofluorescence Microscopy

Thin tissue sections [5 μm] were deparaffinized, dehydrated, and then rehydrated using a standard method [26]. After blocking the sections with albumin, antigens were retrieved by exposing them to microwaves. Primary and secondary antibodies were used in appropriate dilutions. Claudin isoforms -1, -2, -3, and -4 were stained in the tissue sections using fluorescent-labeled 2° Ab-FITC conjugates. Nuclei were counterstained with 60 nM DAPI. Finally, the slides were washed with 1xPBS and mounted with a Vectashield antifade mounting medium [Vector laboratories, Newark, CA, USA]. Cover slips were placed over the tissue sections and sealed with nail polish. The sections were analyzed and photographed using a confocal microscope [ZEISS LSM 510 META, Berlin, Germany].

### 2.11. ECL Western Blot Analysis

Colonic tissues were chopped finely with scissors using a 4-morpholinepropanesulfonic acid buffer, pH 7.4, homogenized, and centrifuged at 600× *g* for 10 min at 4 °C. The supernatants were passed through a cheese cloth and recentrifuged at 5000× *g* for 10 min at 4 °C, and the supernatants so obtained were used for measuring claudin protein isoforms by ECL Western blot analysis [28,29]. A portion of each supernatant was centrifuged at 188,000× *g* for 45 min at 4 °C [Sorvall, Dumfries, UK] to prepare crude microsomes. Total protein concentrations in the lysates and crude microsomal samples were measured using a protein dye-binding assay kit [BioRad, Hercules, CA, USA]. The loading samples [2–3 mg/mL] were prepared and electrophoresed on an 8% polyacrylamide separating gel along with a pre-stained protein size marker [Biorad, Dumfries, VA, USA] at 60 volts for 1–2 h at an ambient temperature. Subsequently, the separated proteins were electrically transblotted from the gel onto PVDF membranes [GE Healthcare, Amersham-Hybond TM-P, Zürich, Switzerland]. The membranes were washed thoroughly with 1xPBS and blocked with 5% skimmed milk solution for 30 min. The membranes were incubated with appropriate primary antibodies [Santa Cruz Biotechnology, Dallas, TX, USA] followed by a peroxidase-conjugate secondary antibody [AffiniPure goat anti-rabbit IgG, Jackson, MS, USA] for 2 h [29]. All steps were performed at an ambient temperature with gentle shaking. Protein bands were developed using ECL reagents [BioRad, Hercules, CA, USA], detected on a BioRad Chemi Doc [BIO-RAD chemi-DocTM MP Imaging System, Hercules, CA, USA], and quantified using Image Lab software [BioRad, Hercules, CA, USA].

The previously bound antibodies were also stripped off the PVDF membranes by washing them with a buffer containing 62.5 mM tris-base, pH 6.8, 2% SDS, and 100 mM ß-mercaptoethanol for 20 min at 50 °C with constant shaking, and the membranes were reprobed with different antibodies.

### 2.12. mRNA Expression

Total RNA was extracted from colonic segments using a TRIzol kit [Invitrogen, Paisley, UK]. Briefly, tissues were chopped finely in TRIzol solution and homogenized to obtain lysates. The lysates then were extracted with phenol and chloroform, and centrifuged for 10 min at 14,000 rpm. The aqueous layer was collected and mixed with isopropanol to precipitate the total RNA which was recovered as a pellet through centrifugation at 4 °C for 10 min. The RNA pellets were washed with 70% ethanol, air-dried, and suspended in RNase-free autoclaved–deionized water. The concentration and quality of the total RNA were assessed by measuring OD at 260 nm and 280 nm using a spectrophotometer.

It is important to emphasize that DSS was found to inhibit the RT-PCR; therefore, the total RNA extracted using TRIzol kit was further purified by two rounds of precipitation using an 8M LiCl solution [30]. The purified RNA was finally precipitated using 70% ethanol and 3M sodium acetate, pH 5.2, mixed in solution [30]. The yield of total RNA [μg per mg tissue] was estimated. Purified total RNA samples [1 mg/mL aliquots] were stored at −80 °C for later use.

We confirmed the inhibition in the RT-PCR by amplifying β-actin mRNA using β-actin-selective primers [Table 1]. In the purified RNA samples, mRNA was quantitated using an endpoint RT-PCR method; the level was expressed as ratio of claudin mRNA:β-actin mRNA. These results were further validated using a SYBR green RT-PCR method and the level was expressed as 2^−∆∆CT^ following the method described earlier [31].

## 3. Statistical Analysis

The data are presented as the mean ± SE. Two-way analysis of variance (ANOVA) was used to test the difference between means of more than two groups. The significance was further evaluated with a post hoc Tukey test using GraphPad Prism. A non-parametric, unpaired, two-tailed *t*-test was performed using Microsoft excel, and a value of significance of *p* < 0.05 was considered statistically significant compared with the respective controls.

## 4. Results

### 4.1. Nobiletin Treatment Attenuates DSS-Induced Colitis

**Figure 1:**
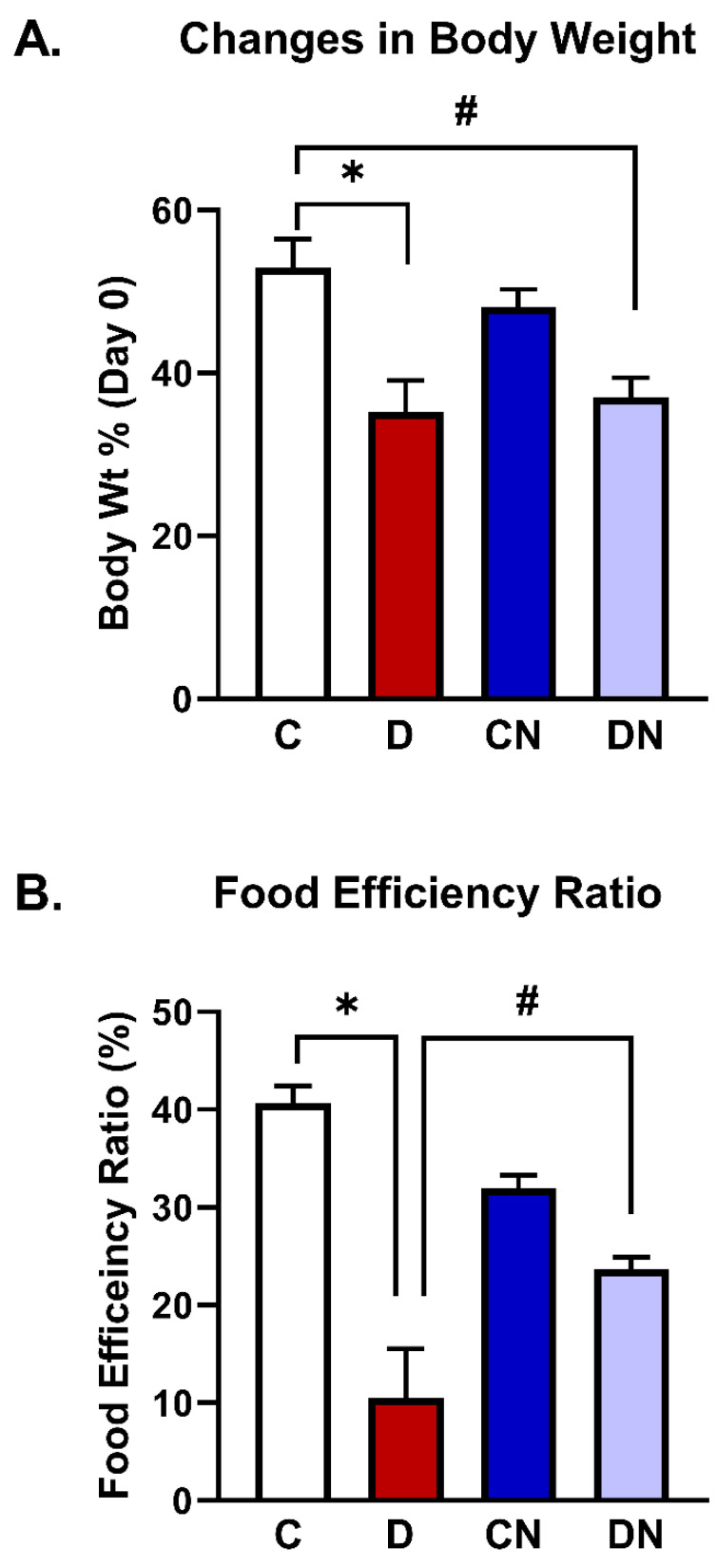
(**A**) Body weight (Wt) change [percentage of day 0 body weight] and (**B**) the food efficiency ratios of the animals from the test conditions: C [non-colitis control], D [DSS-induced colitis], CN [nobiletin-treated non-colitis control], and DN [nobiletin-treated DSS-induced colitis]. The data are the mean ± SE [n = 10]. *, ^#^ indicate significance at *p* < 0.05 between the indicated pair of groups.

The non-colitis control group gained a significant amount of body weight over the seven-day test period compared to the DSS-induced colitis group [Figure 1A]. There was no significant difference in body weight gain in the colitis group compared to the nobiletin-treated colitis group [Figure 1A].

The food efficiency ratio was significantly reduced in the colitis animals compared to the non-colitis controls. The reduced food efficiency ratio was significantly reversed in the nobiletin-treated DSS animals [Figure 1B].

**Figure 2:**
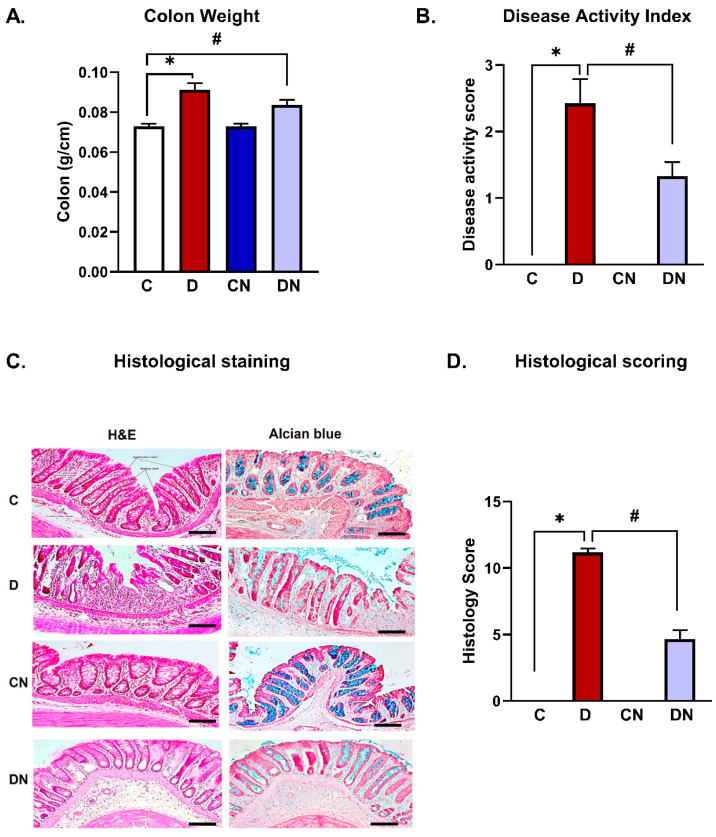
(**A**) Colon weights [g/cm colon length], (**B**) the disease activity score, (**C**) a representative histology of hematoxylin and eosin-stained [left panel] and alcian blue-stained [right panel] colonic tissue sections, and (**D**) the histological score of the animals from the test conditions: C [non-colitis control], D [DSS-induced colitis], CN [nobiletin-treated non-colitis control], and DN [nobiletin-treated DSS-induced colitis]. The data are the mean ± SE [n = 10]. *, ^#^ indicate significance at *p* < 0.05 between the indicated pair of groups. Magnification: 10×. Scalebar = 100 μm.

The progression of colitis was further supported by enhanced colon weight [Figure 2A] and the increased disease activity index in the colitis group compared to the non-colitis controls [Figure 2B]. Nobiletin treatment did not reduce colon weight [Figure 2A]; however, it significantly decreased the disease activity index compared to the colitis group [Figure 2B].

Histologically, the inflamed colons showed epithelial erosion, a depletion of goblet cells, and enhanced infiltration of inflammatory cells into the mucosa compared with the non-colitis control group [Figure 2C,D]. Mucin staining with alcian blue showed a significant reduction in mucin expression in colitis animals compared with the non-colitis controls irrespective of nobiletin-treatment [Figure 2C,D].

**Figure 3:**
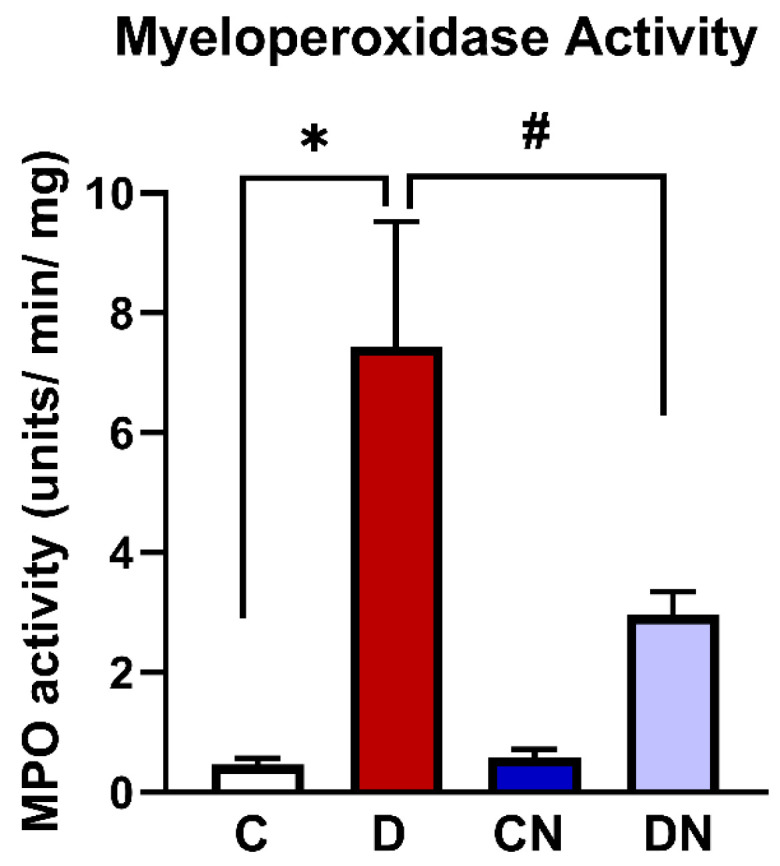
A bar diagram showing MPO activity units/min-mg in the colonic segments from the test conditions: C [non-colitis control], D [DSS-induced colitis], CN [nobiletin-treated non-colitis control], and DN [nobiletin-treated DSS-induced colitis]. The data are the mean ± SE [n = 10]. *, ^#^ indicate significance at *p* < 0.05 between the indicated groups.

In addition, colonic MPO activity was significantly increased in the inflamed colons compared to the non-colitis control group [Figure 3]. MPO activity, however, was significantly reduced in the nobiletin-treated DSS group [Figure 3].

### 4.2. Claudin Expression and Localization

We used two approaches including immunofluorescence microscopy and ECL Western blot analysis to examine quantitative changes in the expression level of claudin isoforms -1, -2, -3, and -4 in rat colons.

**Figure 4:**
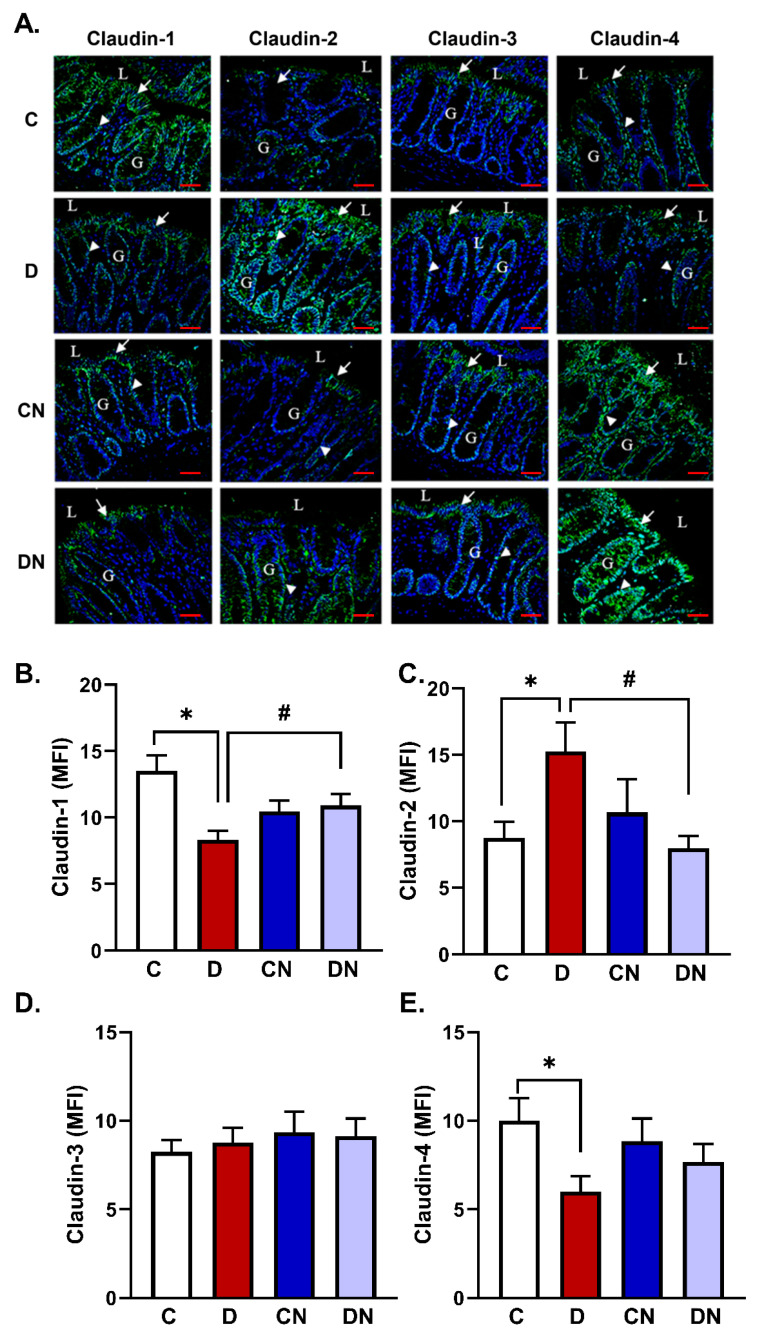
(**A**) Representative photomicrographs showing immunofluorescence staining, and (**B**–**E**) a bar diagram showing the expression levels of the indicated claudin isoforms measured as mean fluorescence intensity [MFI, arbitrary units] using a specific primary antibody and an FITC-labeled secondary antibody in the tissue sections of colon from the test conditions: C [non-colitis control], D [DSS-induced colitis], CN [nobiletin-treated non-colitis control], and DN [nobiletin-treated DSS-induced colitis]. The tissue sections were counter-stained with DAPI. Claudins are expressed in the surface lining epithelium [arrows] and in the epithelial cells [arrow heads] lining the glands [G]. L—lumen of the colon. Magnification = 20×, Scalebars = 100 μm. The data are the mean ± SE [n = 10]. *, ^#^ indicate significance at *p* < 0.05 between the indicated pair of groups.

Immunofluorescence microscopic quantitation and localization demonstrated that the expression of claudin-1 was significantly decreased in the colitis group compared to the non-colitis controls [Figure 4A,B]. In the uninflamed colon, claudin-1 was expressed on the surface epithelium and in the epithelial lining of the glands [Figure 4A]. In the DSS group, claudin-1 expression was decreased on the surface epithelium and in the epithelial lining of the glands [Figure 4A]. In the nobiletin-treated non-colitis group, claudin-1 was predominantly expressed on the surface epithelium and in the epithelial lining of the superficial parts of the glands [Figure 4A]. However, in the nobiletin-treated DSS group, claudin-1 expression was mainly restricted to the surface epithelium and in a small number of glandular cells [Figure 4A] and was significantly increased relative to the colitis group [Figure 4B].

The expression of claudin-2 was significantly increased in the DSS-induced colitis group compared to the non-colitis control group, with extensive expression in both the surface and glandular epithelium [Figure 4A,C]. Claudin-2 expression, however, was reduced in the control and nobiletin-treated groups on the surface epithelium and in the glands [Figure 4A,C]. Moreover, nobiletin treatment of the colitis group significantly reduced claudin-2 protein levels compared to the untreated colitis group, with localized expression in the lining glandular epithelium [Figure 4A,C].

Claudin-3 localized mostly to the surface epithelial cells and the proximal part of the glands. A small number of cells, however, expressed claudin-3 in the deeper parts of the glands in all groups [Figure 4A]. The level of the claudin-3 protein remained unchanged in the experimental groups [Figure 4A,D].

Finally, claudin-4 isoform expression was significantly decreased in the colitis group compared to the untreated controls [Figure 4A,E]. Nonetheless, it was not significantly reversed by nobiletin treatment [Figure 4A,E].

**Figure 5:**
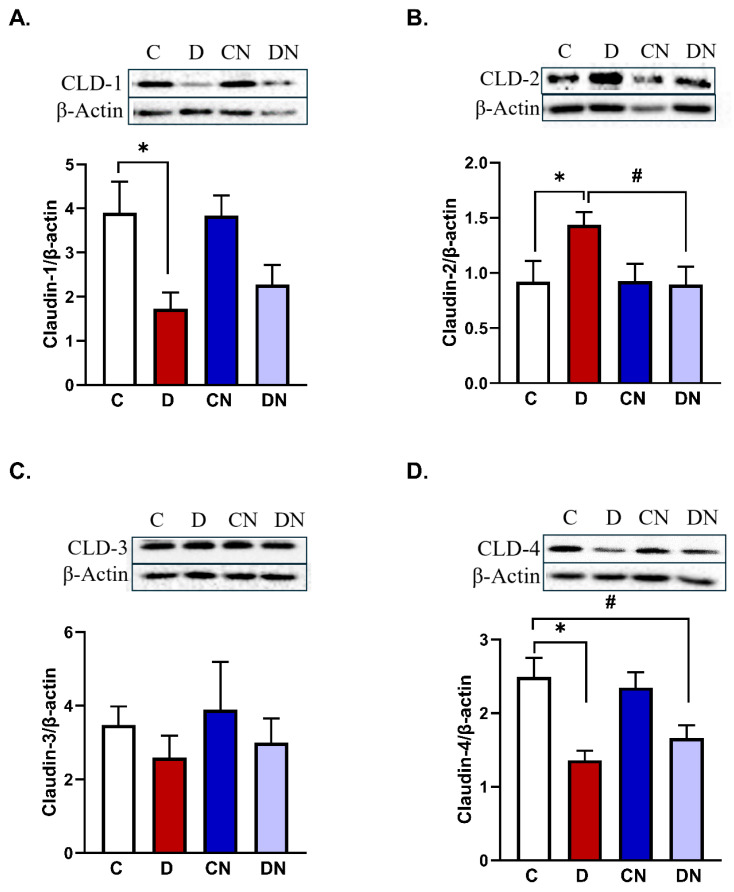
(**A**) A bar diagram showing the protein expression levels relative to β-actin [ratios] of claudin-1, (**B**) claudin-2, (**C**) claudin-3, and (**D**) claudin-4 isoforms in the colon from the test conditions: C [non-colitis control], D [DSS-induced colitis], CN [nobiletin-treated non-colitis control], and DN [nobiletin-treated DSS-induced colitis]. The data are the mean ± SE [n = 10]. *, ^#^ indicate significance at *p* < 0.05 between the indicated pair of groups. *p* < 0.05 with respect to C. The insets show the representative ECL Western blot analysis of the indicated claudin isoform and β-actin.

Consistent with our immunofluorescence experiments, immunoblotting data also demonstrated that DSS-induced inflammation significantly reduced the colonic level of the claudin-1 protein [Figure 5A]. Nobiletin treatment of the colitis animals reversed this reduction significantly, restoring the expression of this isoform [Figure 5A].

Claudin-2 protein levels were significantly increased in colitis animals compared to the non-colitis controls regardless of nobiletin treatment [Figure 5B], and nobiletin treatment of the colitis group significantly reduced the expression of the claudin-2 isoform [Figure 5B].

Moreover, claudin-3 expression did not change significantly across all test conditions, indicating the lack of an effect of inflammation or nobiletin on claudin-3 expression [Figure 5C].

Claudin-4 protein levels were significantly reduced in the colitis group compared to the control group [Figure 5D]. Nobiletin treatment of colitis, however, did not reverse colitis-induced reduction in claudin-4 [Figure 5D].

### 4.3. Effects of DSS-Induced Colitis and Nobiletin on Claudin mRNA Expression

Expression of the claudin-1, -2, and -4 protein isoforms was altered in the inflamed colons compared to the non-colitis controls. Therefore, we focused on these isoforms in the subsequent mRNA analysis. The level of claudin-3 protein expression remained unchanged in the test conditions; therefore, the claudin-3 mRNA isoform was not examined in this study. To quantitate the mRNA levels, we first extracted the total RNA from colons and examined its quality and yields from the test conditions [Supplementary Figure S1]. Then, we examined the samples for the DSS inhibition of the RT-PCR by amplifying β-actin mRNA. As indicated by the intensity of the 18s and 28s rRNA bands, the quality of the total RNA from the test conditions was uniform [Supplementary Figure S1, upper panel]. On the other hand, the expression of β-actin mRNA was inhibited in the RT-PCR before LiCl purification [Supplementary Figure S1, middle panel]. Interestingly, this inhibition was removed by LiCl purification, as indicated by amplification of β-actin in all test samples [Supplementary Figure S1, bottom panel]. The yield of the total RNA in all test samples was uniform.

**Figure 6:**
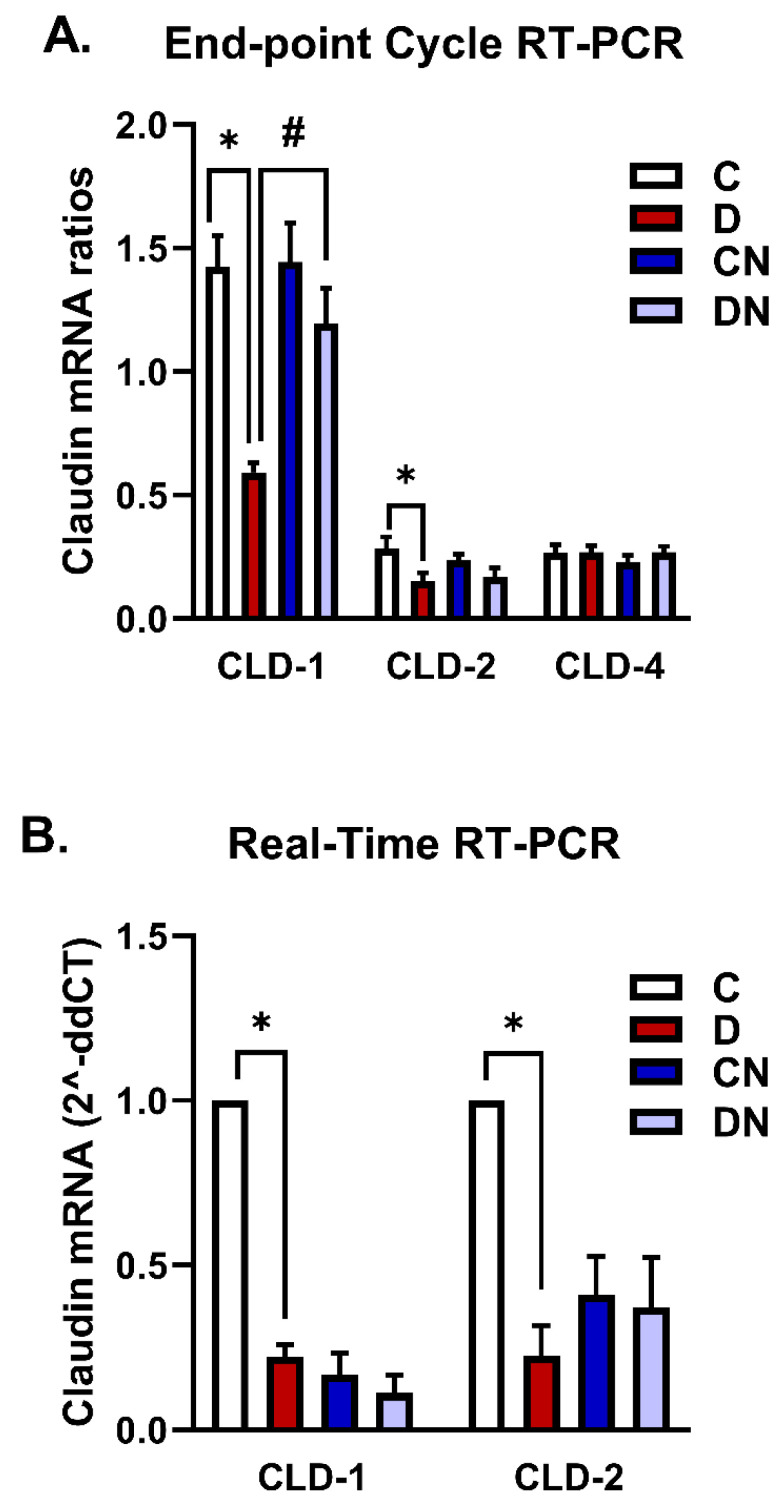
A bar diagram showing the mRNA expression levels as ratios relative to β-actin mRNA of the indicated claudin mRNA isoforms -1, -2, and -4 using (**A**) the endpoint RT-PCR method and (**B**) SYBR green RT-PCR-based C_T_ calculations for claudin isoforms -1 and -2 in the LiCl-purified colonic RNA samples from the test conditions: C [non-colitis control], D [DSS-induced colitis], CN [nobiletin-treated non-colitis control], and DN [nobiletin-treated DSS-induced colitis]. The data are the mean ± SE [n = 10]. *, ^#^ indicate significance at *p* < 0.05 between the indicated pair of groups.

Using the LiCl-purified RNA samples, we estimated the expression level of claudin mRNA relative to β-actin mRNA initially using an endpoint RT-PCR amplification method [Figure 6A]. The levels of claudin-2 mRNA isoforms relative to β-actin decreased significantly compared to the non-colitis controls [Figure 6A]. Nobiletin treatment of colitis did not significantly affect claudin-2 mRNA expression [Figure 6A]. The expression of claudin-4 mRNA remained unchanged in the test conditions [Figure 6A]. These mRNA findings were validated using an SYBR green RT-PCR method. However, since the endpoint RT-PCR data revealed no change in the expression of claudin-4 mRNA in the test conditions, there was no need to validate this by RT-PCR. Therefore, only changes in the level of claudin-1 and -2 mRNA isoforms were validated using the SYBR green RT-PCR method. A similar expression profile of the isoforms -1 and -2 was observed by the SYBR green RT-PCR based on C_T_ calculations [Figure 6B].

## 5. Discussion

IBDs including CD and UC are associated with chronic inflammation in the gastrointestinal tract. Although the etiology of these conditions remains elusive, they are hypothetically produced by an interaction between genetic and environmental factors [32,33]. Consequently, the mechanism of pathogenesis and the treatment for these conditions are not fully established. The key characteristic of these conditions is that they are often diagnosed at a chronic stage, leaving gaps in our understanding of how they initiate and progress from an early state to a full-blown chronic state. Among common IBD conditions, ulcerative colitis is not extensively studied; therefore, this study focuses on its pathogenesis using an experimental model of colitis induced in male Sprague-Dawley rats by administering DSS over seven days. While the DSS-induced colitis model has been extensively used as a model of chronic inflammation in mice, it remains to be incompletely studied how the changes occur at an acute stage in this murine model of colitis. Even in mice, features of IBD related to the pathogenesis reported are inconsistent and variable [34]. Hypothetically, disruption of the intestinal barrier leads to the exposure of lamina propria tissues to microflora, which causes chronic inflammation in the gastrointestinal tract. The main components of the intestinal barrier are a large family of claudin proteins which form a tight junction between the colonic enterocytes.

These claudins are expressed in a cell- and tissue-selective manner. The major claudin isoforms that are expressed in the colon are isoforms -1, -2, -3, and -4. Expression of the claudin isoforms is altered in IBD, but the expression data are inconsistent, lacking uniformity [35,36]. Therefore, further studies are necessary to establish their role in the pathogenesis of IBD. The primary objective of this study was to investigate the roles and mechanism of expression of claudin-1, -2, -3, and -4 isoforms at an early stage of IBD pathogenesis, and to examine the effects of nobiletin on the disease. We used an experimental model of DSS-induced colitis. The groups of colitis rats were also treated daily with an aqueous suspension of nobiletin [60 mg/Kg BW, oral]. We report that nobiletin treatment attenuates DSS-induced colitis, as indicated by an improved disease activity index, histologic score, and FER, along with a reduction in the level of MPO activity and infiltration of neutrophils in the colon.

We utilized well-established macroscopic and microscopic criteria to characterize the colitis. Our findings demonstrated that at a macroscopic level, DSS caused a significant reduction in body weight gain compared to the non-colitis controls or nobiletin-treated non-colitis control. However, decreased body weight gain in colitis did not improve in the nobiletin-treated DSS-induced colitis group. Colitis animals also exhibited a significant decrease in the food efficiency ratio, which was significantly reversed by nobiletin treatment as well. Furthermore, the disease activity index was significantly increased in colitis animals compared to the non-colitis controls, which was also reversed by nobiletin treatment.

Histological findings demonstrated a loss of goblet cells and epithelial cells, erosion of the epithelial cell lining, and a reduction in mucin secretion in the DSS-inflamed colons. Progression of inflammation was also evident from the increased myeloperoxidase activity and the enhanced infiltration of neutrophils into the inflamed colons. The inflamed colons also showed a significant increase in colon weight, further indicating progression of the inflammatory reactions in the present model.

DSS damages the epithelial barrier in the colon; however, the role of claudins, the major component of the epithelial barrier, remains to be established. Encouraged by the observations on colonic biopsies from human IBDs exhibiting reduced electric resistance, many studies have investigated the roles of claudins in the pathogenesis of IBDs [37]. These proteins have been studied extensively and have demonstrated an inconsistency in their expression profiles in models of experimental colitis and in human IBD conditions. Therefore, we examined the roles of claudin-1, -2, -3, and -4 isoforms, which are abundantly expressed in the colon. Claudin-1 and -3 isoforms form a physical barrier, while claudin-2 and -4 isoforms regulate paracellular permeability in the gastrointestinal tract [14,16]. These claudin isoforms are expressed by the colonic enterocytes and form a tight seal or junction against the microflora and foreign antigens [14,16].

To investigate the role and the mechanism of their regulation, we examined the expression of claudin isoforms using immunofluorescence microscopy and immunoblotting techniques. Using both of these approaches, we observed consistently no change in the expression of the claudin-3 isoform in the test conditions, suggesting that it has no role in the development of the acute stage of colitis in the present model. Our findings are, however, different from early reports showing alterations in claudin-3 expression in IBD [5,14,15,16,38]. This discrepancy may be due to differences in the type and the dose of colitogenic substances used, the degree of inflammation produced, and the type of animals used. Interestingly, the expression of the claudin-1 and -4 protein isoforms was reduced in the inflamed colons, suggesting the disruption of colonic tight junction formation, compromising the physical and paracellular permeability barriers which play an important role in the pathogenesis of IBD. On the other hand, claudin-2 was increased, which is expected to increase paracellular permeability, enhancing exposure of the lamina propria to microflora and creating electrolyte disturbance and the production of diarrhea in colitis. It is worth mentioning that earlier findings have reported an increase in the expression of claudin-1 in human colonic biopsies from UC patients which did not correlate with disease severity [39]. Others have reported an increase in the expression of both claudin-1 and -2 isoforms in human IBD [40,41]. Claudin-2 regulates paracellular movement of Na^+^, Ca^2+^, and water in the intestine [42]. Several studies have reported increased expression of claudin-1 and -2 and a reduction in claudin-3 and -4 expression in ulcerative colitis [14]. In Crohn’s disease, upregulation of claudin-1 and claudin-2 and decreased expression of intestinal epithelial isoforms claudin-3, -5, -8, and -12 were reported [9]. These findings thus indicate inconsistency with regard to the role of claudins in the pathogenesis of IBD. Our findings support the reduction in claudin-1 and an increase in claudin-2 expression in inflamed colons. Furthermore, gene knockout studies have suggested a critical role of claudin-3 in maintaining normal microflora and inflammatory responses in IBD [38]. The profile of claudin expression in inflamed colons varies with the types of murine species and strains used [8], but our findings showing no change in the expression of claudin-3 reject a role of claudin-3 in the pathogenesis of acute colitis. Previously, downregulation of claudin-4 and -7, upregulation of claudin-2, and no changes in the expression of claudin-1 and -3 isoforms have also been reported [10]. These findings suggest inconsistent regulation of these proteins in IBD. In conclusion, our immunofluorescence and immunoblotting detection of claudins consistently demonstrates the suppression of claudin-1 and -4, the increase in claudin-2, and no change in the claudin-3 isoform in the present model of ulcerative colitis, all of which are reversed by nobiletin. How these proteins fluctuate in IBD from one study to others has not been addressed. In addition, the molecular mechanism underlying these changes remains incompletely addressed in UC. The role of various mediators has been suggested to upregulate and downregulate the expression of these proteins in a conflicting manner.

We raised that the fluctuations in the protein expression of these isoforms could result from several molecular mechanisms, including transcriptional and post-transcriptional processes. Therefore, in this study, we examined the level of claudin mRNA isoforms in the tissues taken from the test conditions. It is noteworthy that as the claudin-3 protein isoform remained unaltered in the present model, the levels of this mRNA isoform were not investigated any further. Consequently, we focused only on the measurement of the claudin-1, -2, and -4 mRNA isoforms. However, before measuring mRNA levels, we first confirmed that all four selected claudin-1, -2, -3, and -4 mRNA isoforms were expressed in the rat colon. Additionally, each RT-PCR amplified product was also characterized separately by their expected size using DNA size markers [Table 1]. Our findings also demonstrated a uniform quality and yield of the extracted total RNA from the test conditions [Supplementary Figure S1], which rules out any artifacts in the results due to these parameters. Furthermore, since the RT-PCR is an extremely sensitive assay, we therefore co-amplified β-actin simultaneously as an internal control with each claudin isoform. The expression level of claudin mRNA was indicated by a ratio of claudin mRNA:β-actin mRNA. It is also worth mentioning that we observed inhibition of the RT-PCR assay in the samples from D and DN groups. Similar inhibition was attributed to the presence of DSS in the RNA samples by Viennois et al. [30], and it was essential to eliminate DSS from the RNA samples. Therefore, the total RNA samples extracted using the TRIzol kit were purified using a LiCl precipitation method [30] in this study.

For quantitation of the claudin mRNA isoforms, we initially used a classical or endpoint RT-PCR approach, and the data were then validated using a SYBR green RT-PCR method. The endpoint and RT-PCR approaches allow for the quantitation of mRNA accurately in the linear and log phase of amplification, respectively. The endpoint RT-PCR cycle data demonstrated a decrease in the level of claudin-1 and -2 mRNA isoforms, while there was no change in the claudin-4 mRNA level, suggesting differential regulation of these isoforms. Based on a concomitant reduction in the claudin-1 mRNA level, we interpret that claudin-1 is regulated at a transcriptional level in the present model. However, no change in the claudin-4 mRNA level with a decreased protein level suggests post-transcriptional regulation, including translation and a decrease in the stability of the claudin-4 protein. In our study, it is noticeable that the claudin-2 protein level was increased, while its mRNA expression level showed a concomitant reduction, which might be a counteracting mechanism to regulate its expression. While an exact mechanism of regulation remains to be investigated, our findings explain that the suppression of claudin is a complex mechanism regulated post-transcriptionally, including the translation and stabilization of the protein isoform. Nevertheless, these changes in mRNA levels are selective as β-actin expression and the quality and yield of total RNA remained uniform in the test conditions.

We further validated our mRNA findings on claudin-1 and -2 mRNA expression using a SYBR green RT-PCR method. In the RT-PCR method, β-actin was also used as an internal control. Our RT-PCR findings also confirmed the suppression of the claudin-1 and -2 mRNA isoforms.

**Figure 7:**
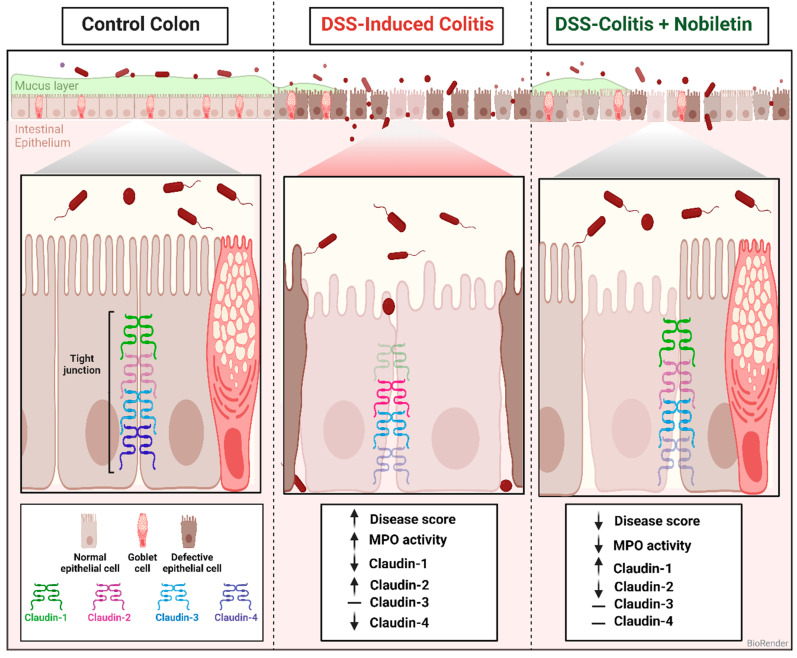
A proposed mechanism of the pathogenesis of DSS-induced ulcerative colitis and its reversal by nobiletin treatment in colitis rats. The figure was created using BioRender with permission.

In conclusion, we demonstrate the suppression of claudin-1 and -4, an increase in claudin-2, and no change in the claudin-3 protein isoform in inflamed colons. These changes are regulated in a complex manner involving both transcriptional and post-transcriptional mechanisms. The altered claudin expression levels are reversed by nobiletin, suggesting that nobiletin may mitigate inflammation by regulating barrier integrity in the present model of colitis. These changes will compromise the formation of a physical and permeability barrier, causing a gut leaky and exposing the lamina propria tissues to luminal microflora, leading to the development of inflammation [Figure 7].

## Figures and Tables

**Table 1:** The upstream [1] and downstream [2] primers for the indicated claudin isoforms 1–4 and β-actin mRNA. The primers’ positions in base pair [bp], the melting temperature (Tm) in °C, the size of the amplified PCR product, and the references used for designing these primers are shown.

Primers	PCR Fragment [bp]	References
1. Claudin-1 5′-GCTTAGAAGATGATGAAGTGCA-3′ [499–520 bp, Tm 62 °C] 2. Claudin-1 5′-CCCACTAGAAGGTGTTGGCT-3′ [781–800 bp, Tm 62 °C]	302 bp	NM_031699
1. Claudin-2 5′-GGTCCCTGACAGCATGAAATT-3′ [801–821 bp, Tm 62 °C] 2. Claudin-2 5′-CACACATACCCAGTCAGGCT-3′ [1021–1040 bp, Tm 62 °C]	240 bp	NM_001106846.2
1. Claudin-3 5′-AGATGGTTACAGACGCCA-3′ [1201–1218, Tm 54 °C] 2. Claudin-3 5′-AAACGGCCCTTTTTATAGTT-3′ [1421–1440, Tm 54 °C]	240 bp	NM_031700.2
1. Claudin-4 5′-CTCAGTCGTAGGGGGCAAG-3′ [482–500, Tm 62 °C] 2. Claudin-4 5′-AAGGCAATGTGGACAGAGAGT-3′ [833–853, Tm 62 °C]	373 BP	NM_001012022.1
1. β-actin 5′-CCTGAGCGCAAGTACTCTG-3′ [2782–2800 bp, Tm 60 °C] 2. β-actin 5′-GTAAAACGCAGCTCAGTAACA-3′ [2920–2940 bp, Tm 60 °C]	159 bp	NC_005111.4

## Data Availability

All data that have been used in the preparation of this manuscript are provided.

## Supplementary Materials

The following supporting information can be downloaded at: https://www.mdpi.com/article/10.3390/biom14091122/s1, Figure S1: ‘RT-PCR inhibition by DSS’.
