# Supplementary figures and images for: The Role of Claudins in the Pathogenesis of Dextran Sulfate Sodium-Induced Experimental Colitis: The Effects of Nobiletin
Source: Biomolecules. 2024 Sep 4;14(9):1122. doi: 10.3390/biom14091122 (PMC11430412; doi:10.3390/biom14091122)

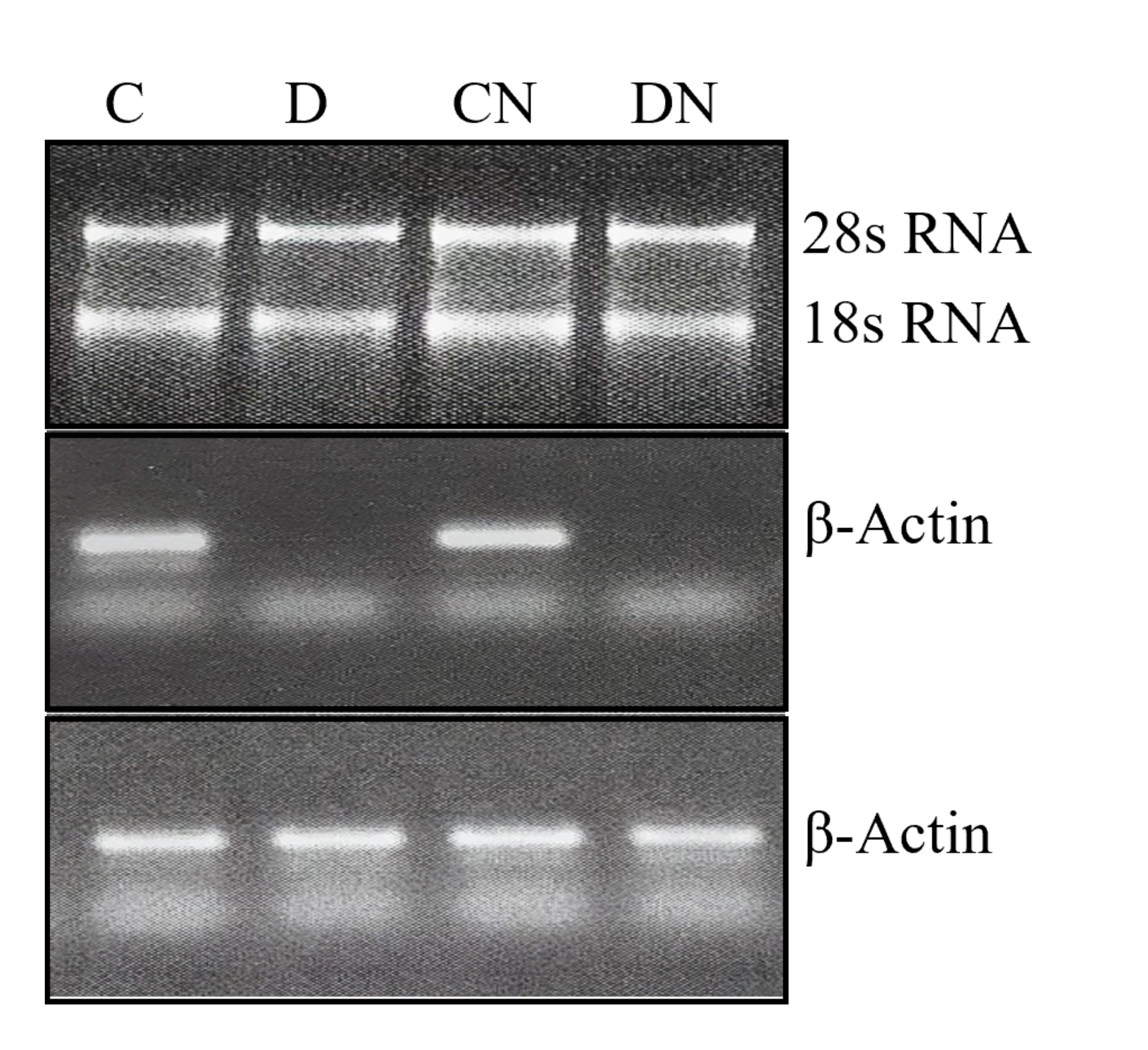

Supplement: Supplementary file 1 [file biomolecules-14-01122-s001.zip › biomolecules-3156983-supplementary.tif]
